# Diffusion tensor imaging metrics associated with future disability in multiple sclerosis

**DOI:** 10.1038/s41598-023-30502-5

**Published:** 2023-03-02

**Authors:** E. Lopez-Soley, E. Martinez-Heras, E. Solana, A. Solanes, J. Radua, F. Vivo, F. Prados, M. Sepulveda, J. M. Cabrera-Maqueda, E. Fonseca, Y. Blanco, S. Alba-Arbalat, E. H. Martinez-Lapiscina, P. Villoslada, A. Saiz, S. Llufriu

**Affiliations:** 1 Center of Neuroimmunology, Laboratory of Advanced Imaging in Neuroimmunological Diseases, Hospital Clinic Barcelona, Institut d’Investigacions Biomediques August Pi i Sunyer (IDIBAPS), Universitat de Barcelona, Calle Villarroel 170, 08036 Barcelona, Spain; 2 Imaging of Mood- and Anxiety-Related Disorders Group, Institut d’Investigacions Biomediques August Pi i Sunyer (IDIBAPS), Universitat de Barcelona, and CIBERSAM, Barcelona, Spain; 3 Centre for Psychiatry Research, Department of Clinical Neuroscience, Karolinska Institutet, Stockholm, Sweden; 4 Early Psychosis Interventions and Clinical-Detection (EPIC) Lab, Department of Psychosis Studies, Institute of Psychiatry, Psychology and Neuroscience, King’s College London, London, UK; 5 E-Health Center, Universitat Oberta de Catalunya, Barcelona, Spain; 6 Centre for Medical Image Computing, Department of Medical Physics and Biomedical Engineering, University College London, London, UK; 7 Queen Square MS Centre, Department of Neuroinflammation, UCL Institute of Neurology, Faculty of Brain Sciences, University College London, London, UK; 8 Department of Neurology, School of Medicine, Pontificia Universidad Católica de Chile, Santiago de Chile, Chile

**Keywords:** Prognostic markers, Multiple sclerosis, Brain, Machine learning

## Abstract

The relationship between brain diffusion microstructural changes and disability in multiple sclerosis (MS) remains poorly understood. We aimed to explore the predictive value of microstructural properties in white (WM) and grey matter (GM), and identify areas associated with mid-term disability in MS patients. We studied 185 patients (71% female; 86% RRMS) with the Expanded Disability Status Scale (EDSS), timed 25-foot walk (T25FW), nine-hole peg test (9HPT), and Symbol Digit Modalities Test (SDMT) at two time-points. We used Lasso regression to analyse the predictive value of baseline WM fractional anisotropy and GM mean diffusivity, and to identify areas related to each outcome at 4.1 years follow-up. Motor performance was associated with WM (T25FW: RMSE = 0.524, R^2^ = 0.304; 9HPT dominant hand: RMSE = 0.662, R^2^ = 0.062; 9HPT non-dominant hand: RMSE = 0.649, R^2^ = 0.139), and SDMT with GM diffusion metrics (RMSE = 0.772, R^2^ = 0.186). Cingulum, longitudinal fasciculus, optic radiation, forceps minor and frontal aslant were the WM tracts most closely linked to motor dysfunction, and temporal and frontal cortex were relevant for cognition. Regional specificity related to clinical outcomes provide valuable information that can be used to develop more accurate predictive models that could improve therapeutic strategies.

## Introduction

Multiple sclerosis (MS) is a chronic inflammatory, demyelinating, and neurodegenerative disease of the central nervous system that can lead to physical and cognitive disability accrual over time^1^. Due to the large variability in disease expression, there is an urgent need to identify those patients presenting faster disability accrual in order to optimise monitoring and treatment management.

MS is characterised by the presence of focal lesions, normal-appearing tissue damage and atrophy, affecting both white matter (WM) and grey matter (GM) integrity, which can be quantified using magnetic resonance imaging (MRI)^2^. Diffusion-weighted imaging (DWI) has enabled a description of microstructural changes related to demyelination and axonal injury in MS, and shows a higher sensitivity and specificity than conventional MRI^3^. The integrity of WM tracts measured by fractional anisotropy (FA) have been linked to concurrent clinical and motor performance, particularly in the corpus callosum and the pyramidal tract^4,5^, as well as cognitive dysfunction, particularly in pathways connecting frontoparietal cortical brain areas, deep GM nuclei and insula^6,7^. Increased mean diffusivity (MD), as a measure of microstructural integrity loss in GM, is also linked to worsening clinical disability^8,9^.

Diffusion tensor metrics combined with demographic, clinical and other neuroimaging variables have been used in a few longitudinal studies to predict disability progression^10^ or cognitive decline^11^, but the specific impact of diffusion metrics on future disability has not yet been fully elucidated, and may differ in motor or cognitive performance. The use of machine learning (ML) techniques enables further study of the predictive value of the diffusion measures, as they can improve complex data analysis. Identifying the specific regions associated with forthcoming disability status may support early recognition of patients with specific pathological damage who will benefit from a more aggressive therapeutic approach^12^, and would also deepen knowledge on the basis of disability evolution. Therefore, the present study aimed to explore the predictive value of the microstructural modifications in WM and GM in mid-term global disability, motor functioning and cognitive performance in MS patients by means of ML techniques. In addition, we further identified those areas that are more closely linked to disability at follow-up.

## Results

We collected data from a cohort of 185 MS patients who attended a baseline and follow-up visit. Median time between visits was 4.1 (range 2.0–8.2) years. In terms of the baseline characteristics (Table 1), the majority of patients were female (71%), middle-aged adults (43 ± 9.7 years), with relapsing–remitting MS (86%) and a median disease duration of 10.6 (range 0.1–41.7) years.

**Table 1:** Demographic and clinical characteristics of MS patients at baseline.

	Median (IQR)
Female, n (%)	131 (71)
Age, mean (SD)	43 (9.75)
Disease duration, median (range)	10.6 (0.1–41.7)
Disease phenotype, n (%)	
Clinically isolated syndrome	12 (6)
Relapsing–remitting multiple sclerosis	158 (86)
Secondary progressive multiple sclerosis	12 (6)
Primary progressive multiple sclerosis	3 (2)
Use of disease modifying therapies, n (%)	101 (55)
Number of previous relapses, median [IQR]	3 [2–5]

The data represent the absolute numbers and proportions of qualitative data, or the median and interquartile range (IQR) for the quantitative data, unless otherwise specified. *SD* standard deviation.

The paired sample t-test used to compare each measure between baseline and follow-up showed a significant difference in T25FW, 9HPT in both hands, and in SDMT (*p* < 0.05), with significantly worse mean scores at follow-up (Table 2). Mean EDSS scores between baseline (range 0–7.0) and follow-up (range 0–8.5) showed no significant differences (*p* = 0.068). During follow-up, 29 (16%) patients experienced increased EDSS. In terms of motor domain, 42 (23%) patients declined in T25FW and 25 (14%) and 17 (9%) patients declined in 9HPT dominant and non-dominant hand respectively. In terms of cognition, 50 (27%) patients worsened in SDMT.

**Table 2:** Disability scores during the study.

	n	Baseline	Follow-up	*p*-value	(95% CI)
EDSS score, median (range)	184	2.0 (0–7.0)	2.0 (0–8.5)	0.068	(− 0.24–0.01)
T25FW, seconds, mean (SD)	185	5.38 (4.95)	6.06 (5.63)	0.004	(− 1.39– − 0.27)
9HPT DH, seconds, mean (SD)	185	20.8 (5.47)	21.8 (7.44)	0.014	(− 2.21– − 0.25)
9HPT NDH, seconds, mean (SD)	185	22 (6.30)	22.8 (6.62)	0.004	(− 2.76– − 0.52)
SDMT score, mean (SD)	185	52.9 (12.8)	50.9 (13.5)	0.017	(0.30–3.09)

*p*-values and 95% confidence intervals (CI) from paired sample t-test to compare outcome scores between baseline and follow-up. The SDMT score was the raw correct number of substitutions.

*EDSS* Expanded Disability Status Scale; *T25FW* Timed 25-Foot Walk; *9HPT* 9 Hole Peg Test; *DH* dominant hand; *NDH* non-dominant hand; *SDMT* Symbol Digit Modalities Test.

One patient was excluded due to an unavailable EDSS score.

## Predictive analysis between DTI metrics and disability outcome

Table 3 summarises the parameters used to evaluate the performance of the association models for each outcome measure. The global disability, measured by the follow-up EDSS, was more closely related to the FA (RMSE = 0.839 and R^2^ = 0.178) than the MD metrics. Similarly, motor functioning was more closely associated with the FA values, both in the ambulation measured by the T25FW (RMSE = 0.524 and R^2^ = 0.304) and in the hand-motor function assessed by the 9HPT (RMSE = 0.662 and R^2^ = 0.062 in the dominant hand, and RMSE = 0.649 and R^2^ = 0.139 in the non-dominant hand). Finally, the predictive cognitive performance model with MD metric (RMSE = 0.772 and R^2^ = 0.186) had better performance than the FA measure. When we compared the model performance metrics for each outcome, we found that they were significantly different (*p* < 0.001; Table 3).

**Table 3:** Performance evaluation of fractional anisotropy and mean diffusivity values predicting each outcome at follow-up.

Outcome	n	DTI measures	RMSE	R^2^	RMSE *p*-value	(95% CI)
EDSS	184	FA	0.839	0.178	< 0.001	(− 0.2, − 0.12)
		MD	0.990	0.038		
T25FW	185	FA	0.524	0.304	< 0.001	(− 0.31, − 0.21)
		MD	0.997	0.012		
9HPT DH	185	FA	0.662	0.062	< 0.001	(− 0.23, − 0.16)
		MD	1.025	0.048		
9HPT NDH	185	FA	0.649	0.139	< 0.001	(− 0.11, − 0.03)
		MD	0.760	0.088		
SDMT	185	FA	0.937	0.098	< 0.001	(− 0.28, − 0.21)
		MD	0.772	0.186		

Results are shown as the Root Mean Square Error (RMSE) and R^2^ value of Lasso models.

Lasso regressions analysis performance. The *p*-values were obtained after comparing the RMSE of the two models for each outcome using a paired sample t-test. The 95% confidence interval refers to the differences in RMSE between the two models. *DTI* diffusion tensor image; *RMSE* Root Mean Squared Error; *R*^2^ R-squared; *CI* confidence interval; *FA* fractional anisotropy of white matter atlas; *MD* median diffusivity from grey matter regions; *EDSS* Expanded Disability Status Scale; *T25FW* Timed 25-Foot Walk; *9HPT* 9 Hole Peg Test; *DH* dominant hand; *NDH* non-dominant hand; *SDMT* Symbol Digit Modalities Test.

One patient was excluded due to an unavailable EDSS score.

## White and grey matter regional analysis

### Regions with significant association between fractional anisotropy measures and disability

**Figure 1:**
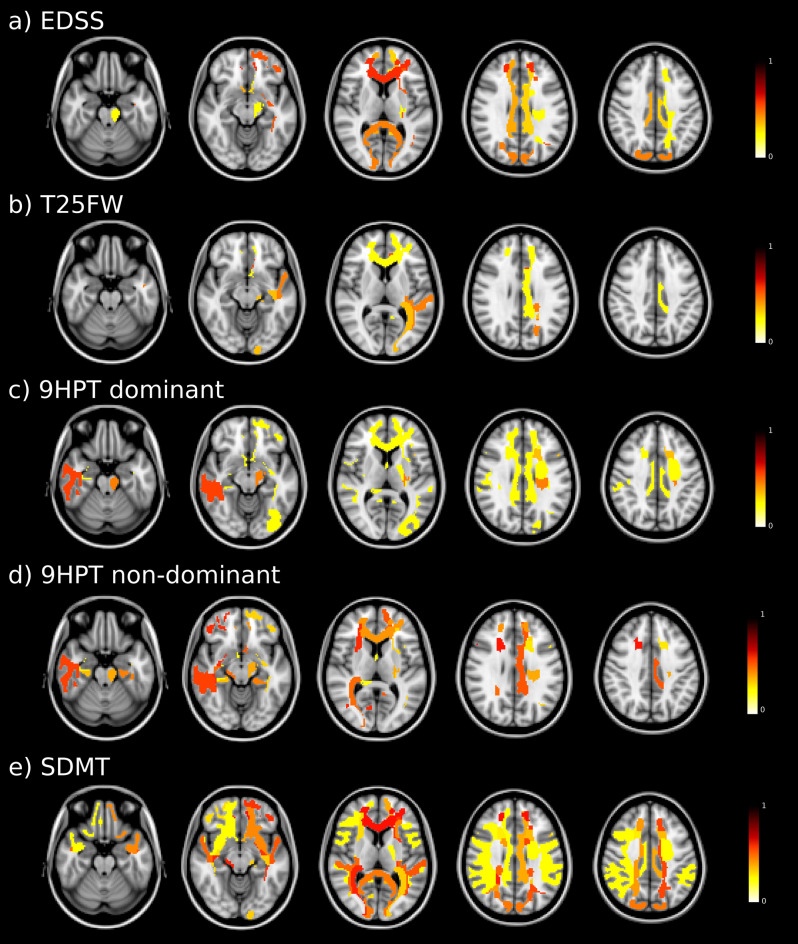
Baseline fractional anisotropy maps associated with follow-up disability outcome. Regions with colours indicate areas of significant associations between white matter fractional anisotropy and clinical outcomes. In the colour scale, 1 represents the maximum coefficient value for the association in each outcome. *EDSS* Expanded Disability Status Scale; *T25FW* Timed 25-Foot Walk; *9HPT* 9 Hole Peg Test; *DH* dominant hand; *NDH* non-dominant hand; *SDMT* Symbol Digit Modalities Test.

Baseline FA in WM tracts were associated with EDSS disability at follow-up, especially those involving the forceps minor and bilateral occipital fasciculus. FA was associated with ambulation, in particular the cingulum, middle longitudinal fasciculus, optic radiation, and forceps minor from the left hemisphere. The upper extremity function was related to FA in the right longitudinal fasciculus, left cingulum and frontal aslant (left hemisphere in the dominant hand, and right hemisphere in the non-dominant hand). Specifically, the 9HPT of the dominant hand at follow-up was also associated with FA in the left corticospinal tract, right fornix, and forceps minor, and in the 9HPT of the non-dominant hand was also related with FA in the occipital fasciculus from the right hemisphere. Finally, the SDMT performance at follow-up was associated with FA, mainly involving the forceps minor, bilateral optic radiation, inferior occipital fasciculus and superior longitudinal fasciculus (Fig. 1).

The bilateral longitudinal fasciculus, inferior occipital fasciculus and the forceps minor were the most relevant WM tracts associated with disability at follow-up in the different clinical measures analysed.

### Regions with significant association between mean diffusivity measures and disability

**Figure 2:**
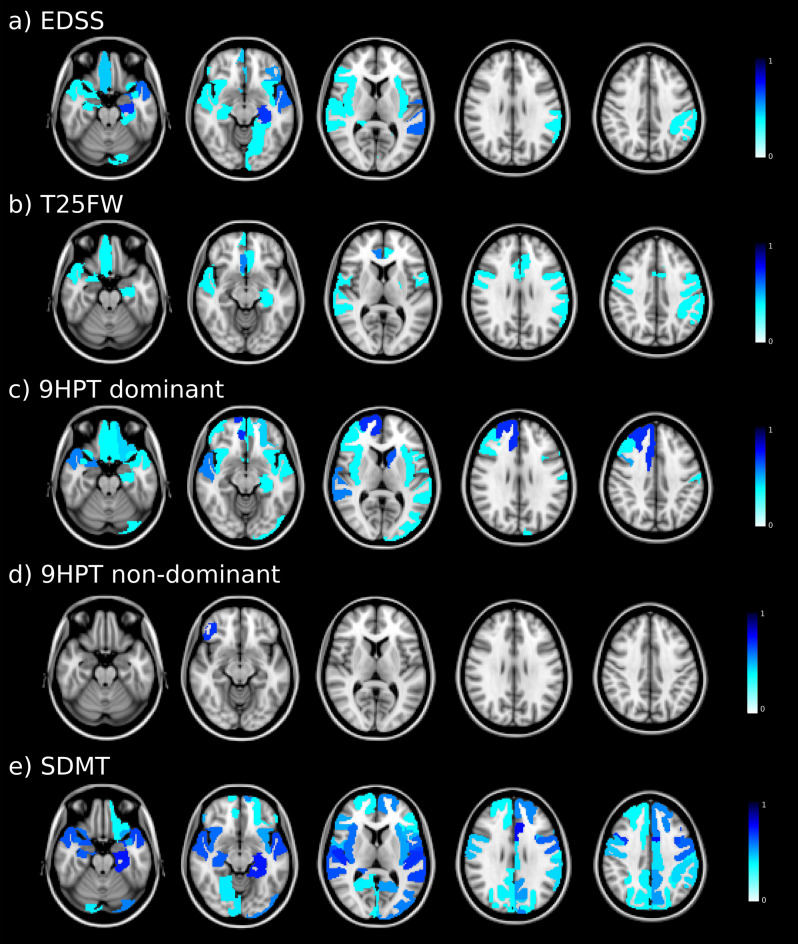
Baseline mean diffusivity maps associated with follow-up disability outcome. Coloured regions indicate areas of significant associations between grey matter mean diffusivity and clinical outcomes. In the colour scale, 1 represents the maximum coefficient value for the association in each outcome. *EDSS* Expanded Disability Status Scale; *T25FW* Timed 25-Foot Walk; *9HPT* 9 Hole Peg Test; *DH* dominant hand; *NDH* non-dominant hand; *SDMT* Symbol Digit Modalities Test.

Based on the relative importance of diffusion metrics, baseline MD values were associated with EDSS at follow-up, especially in areas involving the temporal cortex, such as the hippocampus and superior temporal from the left hemisphere. The T25FW performance at follow-up was related to baseline MD, mainly in the cingulate and frontal lobes, comprising the right rostral anterior cingulate, left precentral and right medial orbitofrontal. At follow-up, 9HPT in both the dominant and non-dominant hand function was associated with baseline MD in the frontal lobe. Areas including the right superior frontal, left caudate, superior temporal and right caudal middle frontal were associated with the 9HPT in the dominant hand, while the right pars orbitalis was the area associated with the 9HPT in the non-dominant hand model. Finally, the SDMT performance was mainly related to MD in temporal and frontal cortex, particularly in areas comprising the left caudal anterior cingulate, bilateral hippocampus, left parahippocampus, and bilateral transverse temporal, caudal middle frontal, and superior temporal (Fig. 2).

Regions from the frontal (bilateral medial orbitofrontal and caudal middle frontal) and the temporal (bilateral superior temporal and left hippocampus) cortex were the most common areas linked to future disability in the various clinical measures analysed.

## Discussion

This study aimed to analyse the predictive value of microstructure diffusion tensor indices and their regional associations with mid-term future disability in patients with MS. Results reveal that at follow-up, global and motor disability is more closely related to WM than GM microstructure, while cognition is mainly associated with GM microstructural properties. Indeed, brain FA in WM tracts explained the 30% and 18% of the variance in future T25FW and EDSS values, respectively, and MD in cerebral GM explained the 18% of SDMT variance. Furthermore, the study found that global disability was associated with integrity of tracts involving forceps minor and occipital fasciculus, and motor performance with cingulum, longitudinal fasciculus, optic radiation, forceps minor and frontal aslant. In contrast, cognitive performance was associated with diffusion changes in the temporal and frontal cortices. These results reinforce the importance of brain microstructural integrity, and highlight the involvement of specific regions in the disability accrual in MS, together contributing to a deeper understanding of the evolution of clinical dysfunction.

Between 9 and 27% of patients in the present study had significant clinical worsening in the various outcomes after a median follow-up of 4 years, with the SDMT showing the largest decline and the 9HTP the lowest. Identifying future clinical status can significantly influence and help tailor pharmacological decision-making. Previous reports have described modifications to diffusion properties that reflect tissue changes related to demyelination, axonal damage and gliosis, not only in visible lesions, but also in normal appearing brain areas, both in WM and GM^4,7,9,13,14^. In MS, microstructural GM changes seem to appear early in the cortex, then extend to subcortical regions, becoming widespread as the disease evolves^8^. Here, we examined their association with future disability using ML techniques that can cope with the complex relationship between clinical and brain impairment, which may not be linear along the course of the illness^15^.

Previous studies analysing the association between MRI data with clinical disability using ML approach used not only diffusion metrics, but also other MRI metrics such as lesion volume and GM volumes as predictors or classifiers^4,12^. In a recent report predicting concurrent EDSS and motor performance with diffusion metrics of motor related tracts, atrophy of sensorimotor areas and functional connectivity of motor hand networks found high predictive value of EDSS (R^2^ = 0.19, MSE = 0.81) specially from atrophy measures and milder prediction for right 9HTP (R^2^ = 0.14, MSE = 0.86) and left 9HTP (R^2^ = 0.24, MSE = 0.76), with the model most influenced by FA in cerebellar peduncle and corpus callosum^4^. Future cognitive decline has been associated (R^2^ = 0.35) with regional MRI measures such as anterior thalamic radiation and superior longitudinal fasciculus FA, together with lesions, temporal cortical volume and age^11^. Results from these models showed that variables such as GM volume or WM lesion volume had the greatest contribution to clinical progression, confirming that widespread structural damage and irreversible tissue loss are associated with severe clinical disability in patients with MS^4,12^. However, disrupted diffusivity measures, which may reflect more premature damage than tissue loss, also contributes to clinical deterioration. Our study evaluated the predictive value exclusively of diffusion metrics in WM and GM and provided the relevant regions associated with each clinical outcome. Results showed that global and motor disability were related to changes in FA values for WM, while cognitive performance was mainly associated with MD metrics for GM. The models assessing ambulation and motor hand performance prediction had the widest discrepancy between the predictive value for each tissue type, which highlights the contribution of brain WM tract changes to motor disability in MS. However, the predictive performance of the different models had an RMSE between 1.025 and 0.524, reflecting the modest predictive value of these models. These results suggest that microstructural changes, which appear earlier than atrophy^8^, are related with the evolution of disability. However, to improve their predictive power, models should include a more comprehensive vision of the disease such as demographic and clinical characteristics, as well as other MRI biomarkers.

When we analysed regional associations, global disability at follow-up was associated with the interhemispheric, cingulum and corticospinal tracts. Likewise, ambulation and hand-motor performance at follow-up were mainly related to the microstructure of the longitudinal fasciculus and cingulum. These results reinforce the contribution of pathways connecting an extensive range of areas related to the motor function of the frontal to parietal cortex, various nodes of the limbic circuitry and the primary visual cortex. These tracts are therefore relevant for tasks implying motor activity and hand–eye coordination. Furthermore, ambulation was associated with the left somato-motor cortex. Conversely, the information processing speed and attention assessed by the SDMT, mainly related to microstructural properties in the cortex and medial temporal regions, highlights the important role these multimodal areas play in maintaining high-performance cognitive functioning^13,16^. Moreover, our results draw attention to the superior longitudinal fasciculus and the optic radiations, which link regions such as frontoparietal lobes and thalamus with the visual cortex, respectively. Both have been related to attention and processing speed-demanding cognitive performance in MS patients, and changes in these long-range connections may promote cognitive dysfunction through a disconnection phenomenon in the structural network^17,18^. All in all, the present findings emphasise the relevance of an optimal large-scale brain network organisation to maintain both motor function and cognitive behaviour in MS patients.

This study has several strengths. The study includes the analysis of microstructural changes in both WM and GM, which could reflect premature damage. In addition, several disability outcomes were analysed, including global, motor, and cognitive data, which provides a comprehensive picture of the functional disability in the disease. Our study had several limitations. The first lies in the fact that working with a real-world MS cohort implies predominantly relapsing–remitting MS patients. This is the most common phenotype encountered clinically in the current treatment era, but is also where disease evolution prediction has scope for more treatment adjustments. Secondly, we used the SDMT, a gold standard tool widely used in MS to assess information processing speed, however it cannot evaluate general cognition. Thirdly, the Lasso regression model could make it difficult to interpret the feature importance because some highly correlated variables may randomly suppress, and their feature weight is reduced to zero. However, it is a useful tool to deal with data sets with potential multicollinearity, and to avoid overfitting in order to obtain more accurate predictions for the regression models. Finally, we had no advanced imaging measures from the spinal cord and cerebellum, and this may have affected the performance of ML motor functioning models, limiting their prognostic accuracy. In further studies, more sophisticated advanced diffusion MRI techniques (e.g. Diffusion kurtosis imaging or multi-compartment biophysical models) would be also investigated to increase the biological specificity of these findings. In addition, the low number of patients with disability worsening limited the application of classification analyses or the regression analysis for each group, which could be addressed in upcoming studies with larger follow-up.

In conclusion, the quantitative diffusion tensor imaging measures were related to mid-term future disability in different tissue types. Results showed regional specificity regarding disability outcome, thus providing relevant information for developing more accurate predictive models that could help improve therapeutic strategies.

## Methods

### Participants

For this longitudinal study, we collected data from a prospective cohort of MS patients diagnosed according to the 2017 McDonald criteria^19^ and recruited at the MS Unit of the Hospital Clinic Barcelona^7,20^. The patients selected and analysed had an MRI scan at baseline and neurological, cognitive, and physical follow-up examinations at different time points of the disease. The inclusion criteria were age between 18 and 65 years, absence of relapses or corticosteroid treatment in the previous month, and stable disease modifying treatment. As such, 185 MS patients fulfilled the inclusion criteria and were included in the analysis. The Ethics Committee at the Hospital Clinic of Barcelona approved the study, and all participants signed an informed consent form prior to inclusion. All study procedures were performed in accordance with the relevant guidelines and regulations.

### Physical and cognitive assessment

This study was designed with continuous overall disability, physical and cognitive scores at follow-up as primary outcome measures. In addition, and for descriptive purposes, participants were categorised as stable or declining for each measure. Overall disability was evaluated by the Expanded Disability Status Scale (EDSS)^21^ score, and disability worsening was defined as an increase in EDSS of 1.5 points, ≥ 1.0 or ≥ 0.5 points in the case of baseline EDSS score of 0, ≤ 5.0, or > 5.0, respectively^22^. Ambulatory function was assessed using the timed 25-foot walk (T25FW) averaged over two consecutive trials, and upper extremity function and dexterity were evaluated by the 9-hole peg test (9HPT), performed twice in each hand. An increase in time of ≥ 20% in both measures was used to define a clinically meaningful disability worsening^23^. Finally, cognition was assessed using alternate versions of the Symbol Digit Modalities Test (SDMT)^24^. This test measures information processing speed, sustained and divided attention, and semantic and working memory, and the decline was defined as a ≥ 10% reduction in the total score^25^.

### Magnetic resonance imaging

#### MRI acquisition protocol

To acquire brain images, we used a 3 T Magnetom Trio scanner (SIEMENS, Erlanger, Germany) with a 32-channel phased-array head coil. For part of the cohort (n = 122 participants; 9% CIS, 82% RRMS and 9% SPMS), MRI scans were acquired according to the following protocol: (1) Three-dimensional Magnetization-Prepared Rapid Acquisition with Gradient Echo (3D-MPRAGE) [TR = 1800 ms; TE = 3.01 ms; TI = 900 ms; 240 sagittal slices with 0.94 mm isotropic voxel size and a 256 × 256 matrix size]; (2) Three-dimensional Fluid Attenuated Inversion Recovery (3D-T2 FLAIR) [TR = 5000 ms; TE = 304 ms; TI = 1800 ms; 192 sagittal slices with 0.94 mm isotropic voxel size and a 256 × 256 matrix size] and (3) the DWI acquisition [TR = 14,800 ms; TE = 103 ms; 100 contiguous axial slices; 1.5 mm isotropic voxel size; 154 × 154 matrix size; b value = 1000 s/mm^2^; 60 diffusion encoding directions and a single baseline image acquired at 0 s/mm^2^]. The remaining participants (n = 63; 2% CIS, 92% RRMS and 6% PPMS) underwent MRI acquisition using the same modality images, but slightly different acquisition parameters: (1) TR = 1970 ms; TE = 2.41 ms; TI = 1050 ms; 208 sagittal slices with 0.9 mm isotropic voxel size and a 256 × 256 matrix size; (2) TR = 5000 ms; TE = 393 ms; TI = 1800 ms; 208 sagittal slices with 0.9 mm isotropic voxel size and a 256 × 256 matrix size and (3) TR = 12,600 ms; TE = 112 ms; 80 contiguous axial slices; 2 mm isotropic voxel size; a 120 × 120 matrix size; b value = 1500 s/mm^2^; 70 diffusion encoding directions and a single baseline image acquired at 0 s/mm^2^.

In addition, field map images were generated to correct any distortions caused by field inhomogeneities (TE 1/TE 2 = 4.92/7.38 ms, with the same slice prescription, slice thickness and field of view according to the DWI sequence).

### Anatomical and diffusion tensor MRI data processing pipelines

In all patients, MS lesions were delineated semi-automatically in the 3D-MPRAGE and registered 3D-FLAIR images using the JIM7 software (Xinapse Systems, Essex, UK) by a trained specialist. Accordingly, the WM lesions were refilled on 3D-MPRAGE in order to improve brain tissue segmentation and registration methods on pathological tissue^26^. Firstly, we assigned 62 cortical GM regions on the lesion-filled 3D-MPRAGE image using Mindboggle-101 according to the Desikan-Killiany-Tourville (DKT) parcellation atlas^27^. Then, 14 subcortical GM structures were automatically extracted using the FSL-FIRST tool^28^. Finally, the refilled 3D-MPRAGE images were normalised to the Montreal Neurological Institute (MNI) space using non-linear symmetric normalisation (SyN) algorithm using the ANTs software package^29^ in order to anatomically define the 42 major WM tracts derived from XTRACT atlas (https://fsl.fmrib.ox.ac.uk/fsl/fslwiki/XTRACT)^30^.

DWI pre-processing and FUGUE fieldmap unwarping frameworks were accomplished using a combination of FSL and MRtrix software packages, as previously described by^31,32^. In addition, diffusion tensor imaging (DTI) scalar maps (FA and MD) were computed using least-squares fitting by FSL’s DTIFIT^33^. Subsequently, GM and WM atlases were adjusted to each DTI space by applying the boundary-based registration inverse transformation matrix obtained from the undistorted EPI images to the 3D-MPRAGE^34^, and the inverse deformation fields obtained from the 3D-MPRAGE image registration to MNI space for the XTRACT atlas. In order to reduce partial volume contamination from the surrounding tissues, all WM and GM masks were eroded by 1 mm. Finally, we computed the mean DTI values within the selected GM and WM regions and harmonised these values with the ComBat function to reduce inter-site variability related to the use of two different acquisition protocols^35,36^.

### Statistical analysis

Descriptive data were presented as median and interquartile range (IQR), or mean and standard deviation (SD) for quantitative variables, as appropriate, and using absolute numbers and proportions of the qualitative variables. The normality assumption was checked by inspecting histograms and using the Shapiro-Wilks test. For descriptive purposes, clinical worsening was described using the paired sample t-test, which evaluates differences between baseline and follow-up of each outcome, following the clinical definitions of significant decline for each measure described above.

We used Lasso regression analysis to explore the predictive value of brain diffusion tensor metrics on overall disability (EDSS score), and physical (T25FW and 9HPT) and cognitive decline (SDMT score) in MS patients at follow-up. The Lasso regression model applies a penalty to variables, resulting in good predictive performance in data sets with potential multicollinearity like this study^37^. We considered two different approaches for each outcome, one from WM atlas, and the other for the cortical and subcortical GM regions. Thus, the models included quantitative DTI measures as potential predictors (mean FA and MD values computed for each region) to assess the predictive value of WM and GM microstructural changes for each clinical outcome. We included age and gender as covariates to control for any potential influence on the results. Model performance was evaluated using the average Root Mean Squared Error (RMSE), defined as the root square of the difference between the predicted and original values, which measures the variance of the residuals, and average R-squared (R^2^). The models were compared using a paired sample t-test to assess whether there is a statistically significant difference between the RMSE of the two models.

We used a tenfold cross-validation method to validate the model’s performance, which involved splitting the overall sample into training and test datasets. We fitted the Lasso regression models using only the training datasets, and assessed the predicted performance using the independent test dataset. The same procedure was used for each specific outcome (EDSS, T25FW, 9HPT in both hands, and in SDMT), repeating the cross-validation procedure 10 times to obtain an accurate estimate of model performance. All analyses were performed using R statistical software (version 4.0.5, www.R-project.org).

## Data Availability

The datasets generated and/or analysed in the current study, as well as the code, are available from the corresponding authors upon reasonable request.
